# *Escherichia coli* tRNA (Gm18) methyltransferase (TrmH) requires the correct localization of its methylation site (G18) in the D-loop for efficient methylation

**DOI:** 10.1093/jb/mvad076

**Published:** 2023-10-16

**Authors:** Yoh Kohno, Asako Ito, Aya Okamoto, Ryota Yamagami, Akira Hirata, Hiroyuki Hori

**Affiliations:** Department of Materials Science and Biotechnology, Graduate school of Science and Engineering, Ehime University, 3 Bunkyo-cho, Matsuyama, Ehime 790-8577, Japan; Department of Materials Science and Biotechnology, Graduate school of Science and Engineering, Ehime University, 3 Bunkyo-cho, Matsuyama, Ehime 790-8577, Japan; Department of Materials Science and Biotechnology, Graduate school of Science and Engineering, Ehime University, 3 Bunkyo-cho, Matsuyama, Ehime 790-8577, Japan; Department of Materials Science and Biotechnology, Graduate school of Science and Engineering, Ehime University, 3 Bunkyo-cho, Matsuyama, Ehime 790-8577, Japan; Department of Natural Science, Graduate School of Technology, Industrial and Social Science, Tokushima University, 2-1 Minamijosanjimacho, Tokushima, Tokushima 770-8506, Japan; Department of Materials Science and Biotechnology, Graduate school of Science and Engineering, Ehime University, 3 Bunkyo-cho, Matsuyama, Ehime 790-8577, Japan

**Keywords:** tRNA, RNA modification, tRNA methyltransferase, SPOUT, Escherichia coli

## Abstract

TrmH is a eubacterial tRNA methyltransferase responsible for formation of 2’-*O*-methylguaosine at position 18 (Gm18) in tRNA. In *Escherichia coli* cells, only 14 tRNA species possess the Gm18 modification. To investigate the substrate tRNA selection mechanism of *E. coli* TrmH, we performed biochemical and structural studies. *Escherichia coli* TrmH requires a high concentration of substrate tRNA for efficient methylation. Experiments using native tRNA ^**Ser**^_**CGA**_ purified from a *trmH* gene disruptant strain showed that modified nucleosides do not affect the methylation. A gel mobility-shift assay reveals that TrmH captures tRNAs without distinguishing between relatively good and very poor substrates. Methylation assays using wild-type and mutant tRNA transcripts revealed that the location of G18 in the D-loop is very important for efficient methylation by *E. coli* TrmH. In the case of tRNA^**Ser**^**, tRNA**^**Tyr**^**and tRNA**^**Leu**^, the D-loop structure formed by interaction with the long variable region is important. For tRNA^**Gln**^, the short distance between G18 and A14 is important. Thus, our biochemical study explains all Gm18 modification patterns in *E. coli* tRNAs. The crystal structure of *E. coli* TrmH has also been solved, and the tRNA binding mode of *E. coli* TrmH is discussed based on the structure.

## Graphical Abstract

**Graphical Abstract:**
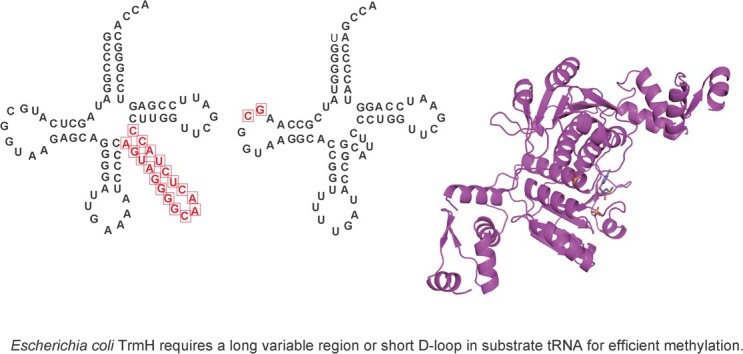

## Abbreviations

Gm: 2’-*O*-methylguanosine
Ψ: pseudouridine

2’-*O*-methylation of ribose is one of the abundant modifications of tRNA *(**1*–*4**)*. Because 2’-*O*-methylation affects the equilibrium of ribose puckering from C2′-endo form to C3’-endo form *(**5**)*, it results in rigidity of local structure in tRNA. Furthermore, because 2’-*O*-methylation prevents cleavage by RNases *(**6**)*, it may contribute to prolong the half-life of tRNA.

Guanosine at position 18 (G18) in tRNA is often modified to 2’-*O*-methylguanosine (Gm18) *(**7**)*. G18 (or Gm18) forms a tertiary base pair with pseudouridine at position 55 (Ψ55) in tRNA and contributes the maintenance of the L-shaped tRNA structure *(**8*–*11**)*. In the case of *Thermus thermophilus*, an extreme thermophilic eubacterium, Gm18 modification stabilizes the tRNA structure at high temperatures through a network between modified nucleosides and tRNA modification enzymes *(**12*–*16**)*. In this network, the presence of 7-methylguanosine at position 46 in tRNA accelerates the formation of Gm18 *(**12**)*. In contrast, the presence of Ψ55 in tRNA suppresses the formation of Gm18 at low temperatures through the network *(**13**)*. In humans, Gm18 modification in tRNA suppresses the immune response through Toll-like receptor 7 *(**17*–*19**)*. Thus, Gm18 modification in human tRNA works as a marker of intrinsic tRNA. Gm18 modification in tRNA from enterobacteria including *Escherichia coli* functions in avoidance of the host immune system *(**17*–*19**)*. Furthermore, recently, it was reported that Gm18 level in *E. coli* tRNAs increased under bacteriostatic antibiotic stress *(**20**)*.

The 2’-*O*-methylation of G18 in tRNA is catalyzed by TrmH in eubacteria *(**21*–*23**)*, Trm3 in yeast *(**24**)* and TARBP1 in humans *(**19**,**25**)*. Recently, Gm18 modification by L7ae, Nop5, archaeal Fibrillarin and Box C/D guide RNA system *(**26**,**27**)* was found in tRNAs from *Pyrococcus furiosus* and *Sulfolobus acidocaldarius (**28**)*. Thus, Gm18 modification in tRNA is common in all three domains of life, eubacteria, archaea and eukaryotes.

S-adenosyl-L-methionine (SAM)-dependent methyltransferases are classified according to the structure of catalytic domain *(**29**)*. Although the majority of tRNA methyltransferases are classified as Class I enzymes, TrmH belongs to the Class IV enzymes *(**29**)*. The Class IV enzymes are known as SpoU-TrmD (SPOUT) superfamily enzymes *(**30*–*32**)* with SpoU being the classical name of TrmH *(**21**,**22**)*. TrmD is a eubacterial tRNA methyltransferase responsible for formation of 1-methylguanosine at position 37 in tRNA *(**33**,**34**)*. Although TrmH and TrmD were previously considered to be unrelated, a bioinformatics study identified that they share three amino acid sequence motifs *(**30**)*. Furthermore, structural studies of TrmH *(**35**)* and TrmD *(**36*–*38**)* have revealed that the catalytic domain of both enzymes contains a deep trefoil-knot structure. Thus, nowadays, TrmH and TrmD are considered to be derived from a common ancestral protein.

TrmH enzymes have been divided into two types according to their substrate tRNA specificities. Type I enzymes such as *T. thermophilus* TrmH can methylate all tRNA species *(**39**)* whereas Type II enzymes such as *E. coli* TrmH *(**7**,**22**)* and *Aquifex aeolicus* TrmH *(**40**,**41**)* methylate only limited tRNA species.

The relationship between function and structure of TrmH has been studied mainly using *T. thermophilus* TrmH. Amino acid residues in the conserved motifs in TrmH are required for formation of the trefoil-knot structure, which forms a SAM-binding pocket and is involved in the catalytic mechanism *(**35**,**42**)*. An arginine residue in motif 1 (Arg41 in *T. thermophilus* TrmH) is essential for the methyltransfer activity *(**35**,**42*–*44**)* and is conserved in all 2’-*O*-methyltransferases in the SPOUT superfamily *(**30**,**31**,**45**)*. Furthermore, basic amino acid residues which mediate tRNA binding have been identified and these residues are conserved in TrmH proteins including *E. coli* TrmH *(**46**)*. Because the methylation site (ribose of G18) is embedded in the L-shaped tRNA structure, the enzyme reaction is composed of at least two steps, namely initial binding and induced fit processes *(**47**)*. Non-substrate tRNA (methylated tRNA) is excluded from the tRNA-TrmH complex by movement within the catalytic domain at the initiation of the induced-fit process *(**48**)*.

The progress described above has been made over the past 20 years. However, one important question remains, namely as to how *E. coli* TrmH selects specific tRNAs given that only 14 tRNA molecular species in 47 present in this eubacterium have the Gm18 modification *(**7**)*. To address this issue, in the current study, we have performed biochemical and structural studies.

## Materials and Methods

### Materials

[Methyl-^3^H]-SAM (2.89 TBq/mmol) and [Methyl-^14^C]-SAM (1.95 GBq/mmol) were purchased from ICN. Non-radioisotope-labeled SAM was obtained from Sigma. DNA oligomers were obtained from Thermo Fisher Scientific. T7 RNA polymerase was purchased from Toyobo. All other chemical reagents were of analytical grade.

### Construction of *E. Coli* TrmH expression vectors

The *trmH* gene was amplified from *E. coli* HB101 strain (Takara) genomic DNA using following primers: EcoTrmHN, 5’-CCC CAT ATG AAC CCA ACA CGT TAT GCA-3′; EcoTrmHC, 5′-CCC CCT CGA GTT ACC CTG CAG CCT GCA TAG T-3′. Underlining highlights the restriction enzyme sites (Nde I and Xho I sites). The amplified DNA was inserted between the Nde I and Xho I sites of pET28a and pET30a expression vectors (Novagene).

### Expression of *E. Coli* TrmH

In pET28a-EcoTrmH, a 6x His tag is fused to the N-terminal region of TrmH. This protein was used only for crystallization. In contrast, in the case of pET30a-EcoTrmH, TrmH is expressed without a tag sequence. In this study, all biochemical experiments were performed using this protein. *E. coli* BL21 (DE3) Rosetta 2 strain (Novagene) was used for the expression. The cells were cultured at 37°C. When the optical density (600 nm) reached 0.8, isopropyl β-D-thiogalactopyranoside (Nacalai Tesque, Japan) was added (final concentration, 1 mM). The cells were further cultured at 37°C for 4 h and then collected by centrifugation at 6500 x g at 4°C for 20 min. The cells were stored at −80°C before use.

### Purification of *E. Coli* TrmH with 6 x his tag for crystallization

Wet cells (0.5 g) were suspended in 7.5 mL buffer A [50 mM Tris–HCl (pH 7.6), 5 mM MgCl_2_, 200 mM KCl, 5 mM imidazole, 5% glycerol] with 75 μL protease inhibitor cocktail (Nacalai Tesque, Japan, code 03969-21), and then disrupted with an ultrasonic disruptor model UD-200 (Tomy, Japan). The cell debris was removed by centrifugation at 8000 x g at 4°C for 20 min. The supernatant fraction was loaded onto a Ni-NTA Superflow column (Qiagen, 5 mL). Fractions containing TrmH were obtained using an imidazole linear gradient (5–500 mM) developed in buffer A and then combined. The sample was loaded onto a HiTrap Heparin HP column (GE Healthcare, 5 mL). TrmH was eluted using a KCl linear gradient (200–1000 mM) in buffer B [50 mM Tris–HCl (pH 7.6), 5 mM MgCl_2_, 200 mM KCl, 5% glycerol]. The fractions were combined and concentrated with a Vivaspin 15R filter device (Millipore, molecular cut-off, 10,000). The concentrated sample (5.94 mg/ml) was directly used for screening of crystallization.

### Purification of *E. Coli* TrmH (without a tag sequence) for measurement of activity

Wet cells (1.0 g) were suspended in 10 mL buffer B supplemented with 100 μL protease inhibitor cocktail, disrupted with an ultrasonic disruptor, and then centrifuged at 8000 x g at 4°C for 15 min. The supernatant fraction was loaded onto a HiTrap Q column (GE Healthcare, 5 mL). TrmH was eluted using a KCl linear gradient (200–1000 mM) developed in buffer B and then loaded onto a HiTrap Heparin HP column (5 mL). Elution was performed with a KCl linear gradient (200 mM–1000 mM) in buffer B. TrmH fractions were combined and dialyzed against buffer B containing 400 mM KCl and 50% glycerol at 4°C overnight. The sample was stored at −30°C.

### Preparation of tRNA transcripts

Wild-type and mutant tRNA transcripts were prepared by *in vitro* T7 RNA polymerase transcription as reported previously *(**49**)*. The transcripts were purified by Q-Sepharose (GE Healthcare) chromatography and 10% polyacrylamide gel electrophoresis in the presence of 7 M urea [10% PAGE (7 M urea)].

### Purification of tRNA^Ser^_CGA_ from *E. Coli trmH* gene disruptant strain

*Escherichia coli trmH* gene disruptant strain (Keio Collection, ID 6663) *(**50**)* was purchased from National Institute of Genetics, Japan. A small RNA fraction (mainly tRNA) was prepared using the acid guanidinium thiocyanate-phenol-chloroform extraction method *(**51**)* and Q-Sepharose column chromatography. Transfer RNA^Ser^_CGA_ was purified using the solid-phase DNA probe method *(**52**,**53**)* using the following primer: 5′- TCG AGA CCG GTC CGT TCA GCC GC –biotin 3′. The eluted tRNA^Ser^_CGA_ was further purified by 10% PAGE (7 M urea).

### Measurement of enzymatic activity

**Fig. 1:**
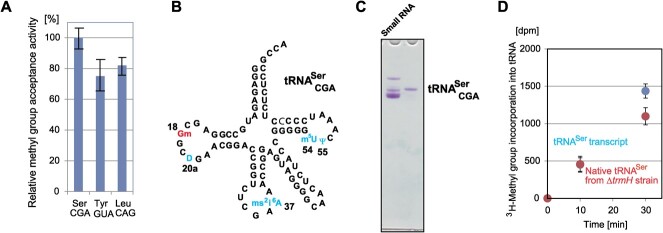
**tRNA** ^**Ser**^_**CGA**_ **is a good substrate for *E. coli* TrmH and the modified nucleosides in native tRNA** ^**Ser**^_**CGA**_ **do not have an effect on the methylation speed.** (A) The relative methyl group acceptance activities of tRNA^Ser^_CGA_, tRNA^Tyr^_GUA_ and tRNA^Leu^_CAG_ transcripts were compared. The methyl group acceptance activity of tRNA^Ser^_CGA_ over a 10 min period is expressed as 100%. (B) The sequence of tRNA^Ser^_CGA_ is depicted as a cloverleaf structure. Numbers show the positions of modified nucleosides. (C) Native tRNA^Ser^_CGA_ was purified from the *E. coli trmH* gene disruption strain. The small RNA fraction (left, 0.30 A260 units) and purified tRNA^Ser^_CGA_ (right, 0.025 A260 units) from the *E. coli trmH* gene disruption strain were analyzed by 10% PAGE (7 M urea). The gel was stained with toluidine blue. (D) The methyl group acceptance activity of purified tRNA^Ser^_CGA_ from the *E. coli trmH* gene disruption strain was compared to that of tRNA^Ser^_CGA_ transcript.

**Fig. 2:**
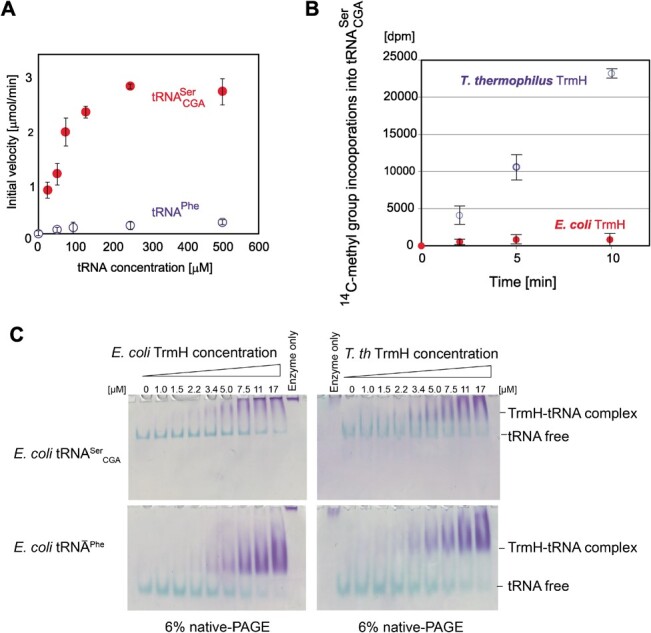
***E. coli* TrmH binds very poor substrate (tRNA** ^**Phe**^ **) as well as good substrate (tRNA** ^**Ser**^_**CGA**_ **).** (A) The initial velocities of methyl-transfer to tRNA^Ser^_CGA_ (filled circles) and tRNA^Phe^ (open circles) by *E. coli* TrmH were measured at various concentrations of tRNA. (C) The results of gel mobility shift assays with tRNA^Ser^_CGA_ (upper) and tRNA^Phe^ (lower) are compared. As positive controls, *T. thermophilus* TrmH were used (right panels). The samples were loaded onto 6% native polyacrylamide gels and the gels were doubly stained with Coomassie Brilliant Blue for detection of protein and methylene blue for detection of tRNA transcript.

**Fig. 3:**
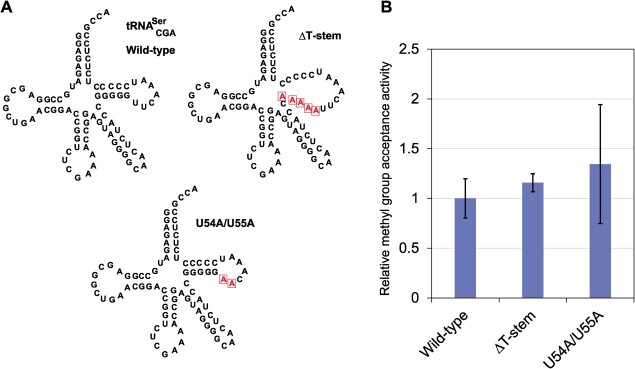
***E. coli* TrmH does not require the interaction between D-loop and T-loop.** (A) Cloverleaf structures of wild-type and mutant tRNA^Ser^_CGA_ transcripts used in this experiment are shown. The mutation sites are enclosed by squares. (B) Methyl-group acceptance activities of the transcripts are compared. The methyl group acceptance activity of wild-type tRNA^Ser^_CGA_ is expressed as 100%.

A standard assay for enzyme activity was performed, in which incorporation of the methyl group from [methyl-^3^H]-SAM into *E. coli* tRNA^Ser^_CGA_ transcript was monitored *(**49**)*. Typical concentrations of SAM and tRNA for the kinetic analyses were as follows: SAM, 200 μM; *E. coli* tRNA^Ser^_CGA_ transcript, 0, 25, 50, 125, 250 and 500 μM. The concentration of SAM was adjusted by mixing of [methyl-^3^H]-AdoMet (0.3 μM) and non-radioisotope labeled SAM (199.7 μM). The concentration of TrmH was fixed at 0.67 μM. The reaction mixture (30 μL) in buffer B was incubated at 37°C for appropriate times (0, 5, 10, 15, 20, and 30 min) and conventional filter assays were performed. For the initial 10 min, linearity of the methyl-transfer reaction was confirmed. In the case of experiments in Supplementary Fig. S1B and C, TrmH (0.5 mg/ml) was stored at 4°C and − 30°C for 14 days, and then 1.0 μM TrmH and 250 μM tRNA^Ser^_CGA_ transcript were incubated in the presence of 200 μM SAM at 37°C for 30 min. In the case of experiments in Fig. 1A, 2A, 3B, 6F and 7D, the initial velocity was calculated from the data at 10 min using 1 μM TrmH. The data are the average of four independent experiments.

### Gel mobility shift assay

Gel mobility shift assays were performed according to our previous report *(**42**)* with slight modifications as follows. 4.25 μM *E. coli* tRNA^Ser^_CGA_ or tRNA^Phe^ was incubated with several concentrations (0, 1.0, 1.5, 2.2, 3.4, 5.0, 7.5, 11.0, 17.0 μM) of *E. coli* TrmH or *T. thermophilus* TrmH at room temperature for 30 min in 60 μL of binding assay buffer [50 mM Tris–HCl (pH 7.6), 5 mM Mg-acetate, 6 mM 2-mercatoethanol, 400 mM KCl, 5% glycerol] and then 20 μL of the samples were used for 6% native gel electrophoresis. The gels were stained with Coomassie Brilliant Blue for detection of protein and then stained with methylene blue for detection of RNA.

### Site-directed mutagenesis

Site-directed mutagenesis was performed using a Quick-Change Mutagenesis kit (Stratagene).

### Crystallization

Crystal screening of the *E. coli* TrmH was performed at 20°C by the hanging-drop vapor diffusion method. However, initial crystallization trial was not succeeded. Therefore, we constructed mutant *E. coli* TrmH (E107G) in which the Glu107 was replaced with Gly and then crystallized the mutant protein. Considering the sequence alignment between the *E. coli* and *T. thermophilus* TrmH based on the crystal structure of *T. thermophilus* TrmH, the Glu107 in *E. coli* TrmH is likely to be located on the molecular surface, which is not close to the SAM-binding site. The negative charge of carboxyl group in Glu107 possibly interferes with crystallization of *E. coli* TrmH. To lose the negative charge, mutant E107G was prepared. A single crystal of the E107G mutant appeared after 1 day in 100 mM HEPES-NaOH buffer (pH 7.6) containing 5% 2-methyl-2,4-pentanediol (MPD) and 10% polyethylene glycol (PEG) 6000. To determine the crystal structure of E107G and SAM complex, co-crystallization of the complex was performed as follows. In 15.3 mg/ml of E107G, SAM was added to a final concentration of 2 mM. 2 μL of the solution was mixed 2 μL of reservoir solution (100 mM HEPES-NaOH (pH 7.6), 5% MPD and 10% PEG 6000). 4 μL of the drop solution was equilibrated against 500 μL of the reservoir solution at 20°C. Full-sized cubic-shaped (300 × 200 × 200 μm) crystals grew within 1 day. SeMet crystals grew under the same conditions.

### Structural determination

The structure of E107G-SAM was refined to *R*_work_/*R*_free_ of 20.2%/24.2% at 1.95 Å resolution (Table 1). The crystal belonged to space group *C*2, where one E107G-SAM molecule is present per asymmetric unit. The final model of E107G-SAM contained 236 residues, 66 water molecules, 1 phosphate ion and 1 SAM molecule. The final model was further checked using PROCHECK *(**54**)*, showing the quality of the refined model. Ramachandran plots (%) of the two structures are tabulated in Table 1. The structure factor and X-ray diffraction data set from E107G-SAM (λ = 1.0000) was recorded at the BL38B1 beamline at SPring-8 (Hyogo, Japan). The SAD data set from SeMet TrmH (λ = 0.9791) was also collected at the same beamline at SPring-8. Data collection statistics are in Table 1. All data sets were processed, merged and scaled using the HKL2000 program *(**55**)*. Using the deduced Se-SAD data set, 10 Se positions were identified and refined in the monoclinic space group *C*2, and the initial phase was calculated using AutoSol in PHENIX *(**56**)*, and followed by automated model building using RESOLVE *(**57**)*. The resulting map and partial model were used for manually building the model using COOT *(**58**)*. The model was further refined using PHENIX *(**56**)*. Using the refined coordinate of SeMet TrmH as a search model, molecular replacement phasing of E107G-SAM crystal structure was achieved using the Phaser program *(**59**)*. The model was further built manually with COOT *(**58**)* and refined with Refmac *(**60**)*. Coordinate of E107G- SAM have been deposited in the Protein Data Bank (PDB code 7EDC).

**Table 1:** Data collection and refinement statistics

	SeMet-E107G	E107G-SAM
*Data collection*		
Space group	*C*2	*C*2
Cell dimensions		
a,b,c (Å)	85.84, 60.26, 68.09	84.79, 60.94, 67.91
α,β,γ (°)	90.00, 123.04, 90.00	90.00, 122.97, 90.00
Resolution (Å)	50 to 2.10 (2.18–2.10)	50 to 1.95 (1.98–1.95)
R_merge_^a^	8.1 (20.8)	4.4 (64.3)
*I/σI*	50.7 (7.5)	47.0 (7.9)
Completeness (%)	99.4 (100.0)	99.6 (99.9)
Redundancy	5.7 (5.7)	3.7 (3.7)
*Refinement*		
Resolution		30.98–1.95
No. reflections		20,209
R_work_^b^/R_free_^c^		20.24/24.21
No. atom		1927
protein		1829
SAM		27 (1 × SAM)
PO_4_^2−^ion		5 (1× PO_4_^2−^ )
water		66
Avg. B-factors (Å^2^)		45.25
R.m.s deviation		
Bond lengths (Å)		0.01
Bond angles (°)		1.64
Ramachandran plot (%)		
Most favored		99.6
Additional allowed		0.4
Generously allowed		0.0
Disallowed		0.0

The value in the parentheses is for the highest resolution shell.

a *R*_merge_ = ΣΣ*_j_*| < *I*(*h*) > − *I*(*h*)*_j_*|/ΣΣ*_j_*| < *I*(*h*) > |, where <*I*(*h*) > is the mean intensity of symmetry-equivalent reflections.

b *R*_work_ = Σ (II*F*_p_(obs) – *F*_p_(calc)II)/ΣI*F*_p_(obs)I.

c *R*_free_ = *R* factor for a selected subset (5%) of reflections that was not included in earlier refinement calculations.

## Results

### A high concentration of KCl stabilizes the activity of *E. Coli* TrmH

*E. coli* TrmH was expressed in *E. coli* cells and purified (Supplementary Fig. S1A). To our surprise, the enzymatic activity of purified TrmH was very unstable and weak. When the purified *E. coli* TrmH was stored at 4°C in the standard store buffer for *T. thermophilus* TrmH [50 mM Tris–HCl (pH 7.6), 5 mM MgCl_2_, 6 mM 2-mercaptoethanol, 50 mM KCl, 5% glycerol] for 14 days, the enzymatic activity was almost lost (Supplementary Fig. S1B). SDS-PAGE analysis revealed that the loss of activity was not caused by proteolysis (data not shown). Furthermore, we observed that a portion of purified TrmH precipitated during the storage. Initially, therefore, we investigated the storage conditions for purified TrmH: the KCl and glycerol concentrations for storage of active *E. coli* TrmH were analyzed (data not shown). As a result, we found that a high concentration (more than 400 mM) of KCl stabilizes the activity of *E. coli* TrmH. When purified *E. coli* TrmH was stored at −30°C in the presence of 400 mM KCl and 50% glycerol for 14 days, the activity did not change significantly (Supplementary Fig. S1C). Based on this observation, we altered the purification procedure, such that KCl was kept at high concentration throughout the purification as described in the Materials and Method. Thus, we were able to obtain *E. coli* TrmH for kinetic studies.

### tRNA^Ser^_CGA_ is a good substrate tRNA for *E. coli* TrmH and modified nucleosides in tRNA^Ser^_CGA_ do not affect methyl group acceptance activity

Next, we determined a model substrate tRNA for kinetic study of *E. coli* TrmH. Of the *E. coli* tRNAs, tRNA^Ser^_CGA_, tRNA^Tyr^_GUA_ and tRNA^Leu^_CAG_ possess the Gm18 modification *(**7**)*. We prepared transcripts of these tRNAs and then compared their methyl group acceptance activities. Figure 1A shows the relative methyl group acceptance activities of these tRNA transcripts at 10 min. Although all these tRNA transcripts were methylated by *E. coli* TrmH, the methyl group acceptance activity of tRNA^Ser^_CGA_ was superior to the others (Fig. 1A). Therefore, we selected tRNA^Ser^_CGA_ as a model substrate. In native tRNA^Ser^_CGA_, four modified nucleosides (D20, ms^2^i^6^A37, m^5^U54 and Ψ55) exist in addition to Gm18 (Fig. 1B). To clarify the effect of these modified nucleosides on the methyl-transfer velocity of TrmH, we purified native tRNA^Ser^_CGA_ from a small RNA fraction of *E. coli trmH* gene disruptant (*ΔtrmH*) strain using a solid-phase DNA probe method (Fig. 1C and ref. *53**)*. The methyl group acceptance activity of purified tRNA^Ser^_CGA_ was compared to that of tRNA^Ser^_CGA_ transcript (Fig. 1D). No significant difference was observed between the transcript and native tRNA from the *ΔtrmH* strain. Thus, the four modified nucleosides (D20a, ms^2^i^6^A37, m^5^U54 and Ψ55) in tRNA^Ser^_CGA_ do not affect the methylation by *E. coli* TrmH.

### Efficient methyl-transfer requires high concentrations of substrate tRNA and non-substrate tRNA (tRNA^Phe^) transcript is methylated slowly at high concentrations

Kinetic parameters for tRNA^Ser^_CGA_ were measured. To our surprise, as shown in Fig. 2A, the apparent *Km* value was very large (90 $\pm$ 10 μM). In contrast, *T. thermophilus* TrnH has a *Km* value for yeast tRNA^Phe^ transcript of around 100 nM *(**39**)*. This is the main reason that the activity of *E. coli* TrmH is very weak. In our standard tRNA methyltransferase assay, tRNA concentration is below 20 μM. To demonstrate the low activity of *E. coli* TrmH, we compared the methylation activities of *E. coli* and *T. thermophilus* TrmH enzymes (Fig. 2B). In this experiment, we intentionally used 50 μM ^14^C-SAM, 20 μM tRNA^Ser^_CGA_ transcript and 0.67 μM *E. coli* or *T. thermophilus* TrmH: this is one of general assay conditions for tRNA methyltransferases. Although the optimum temperature for activity of *T. thermophilus* TrmH is 60–70°C *(**61**)*, *T. thermophilus* TrmH clearly methylated tRNA^Ser^_CGA_ transcript even at 37°C. In contrast, the methylation speed by *E. coli* TrmH was markedly slow. Therefore, hereafter, we used 250 μM (167.0 A260 units/ml) of tRNA transcripts in the assay. This concentration of tRNA is much greater than the amount of native tRNA in living *E. coli* cells. Because *E. coli* tRNA^Phe^ from living cells does not possess the Gm18 modification *(**7**)*, we prepared tRNA^Phe^ transcript as a negative control. Unexpectedly, however, when 250 μM tRNA^Phe^ transcript was used as the substrate, a very slow methyl-transfer reaction was observed (Fig. 2A). We repeated the experiments and confirmed the methylation of tRNA^Phe^ transcript. Although the *Km* value for tRNA^Phe^ transcript could not be accurately measured due to the low methyl group acceptance activity, it is clear that the apparent *Km* value for tRNA^Phe^ transcript is very large.

Because the methylation site (2’-OH of ribose of G18) is embedded in the L-shaped tRNA structure *(**8**,**9**)*, the disruption of L-shaped structure is required for methylation by TrmH. Therefore, the methyl-transfer reaction of TrmH is composed of at least two steps, namely initial binding and induced-fit processes *(**47**,**48**)*. Because the *Km* value is generally calculated from the formation speed of product, both formation of tRNA-TrmH complex and release of products (methylated tRNA and S-adenosyl-L-homocysteine) reflect in the *Km* value. To estimate the affinity of *E. coli* TrmH for tRNA, therefore, a gel mobility shift assay *(**42**)* was performed (Fig. 2C left panels). As controls of gel mobility shift assay, *T. thermophilus* TrmH was used (right panels in Fig. 2C). In these experiments, 400 mM KCl was added into the binding assay buffer for the stabilization of *E. coli* TrmH: 400 mM KCl was also added into the *T. thermophilus* TrmH samples. Even in the presence of 400 mM KCl, the bands corresponding to TrmH-tRNA complex were clearly observed in all gels. The affinities of *E. coli* TrmH for tRNA^Ser^_CGA_ (Fig. 2C left upper) and tRNA^Phe^ (Fig. 2C left lower) were slightly weaker than those of *T. thermophilus* TrmH (Fig. 2C right panels). However, *E. coli* TrmH clearly formed a complex with both tRNA^Ser^_CGA_ and tRNA^Phe^ at concentrations of enzyme below 10 μM. Because the concentrations of tRNA^Ser^_CGA_ and tRNA^Phe^ were fixed at 4.25 μM, the results of gel mobility shift assay reveal that the very large *Km* value (around 90 μM) is not a result of the initial binding process. In the initial binding process, *E. coli* TrmH captures tRNA without distinguishing between relatively good (tRNA^Ser^_CGA_) and very poor (tRNA^Phe^) substrates.

### The basic tRNA recognition mechanism of *E. coli* TrmH is common with that of *T. thermophilus* TrmH

To address the tRNA recognition mechanism of *E. coli* TrmH, we prepared two tRNA^Ser^_CGA_ mutant transcripts (Fig. 3) and tested their methyl group acceptance activities. When the T-stem was disrupted (ΔT-stem), methyl group acceptance activity was retained. Furthermore, disruption of the interaction between T-loop and D-loop (U54A/U55A) did not decrease in the methyl-group acceptance activity. These results clearly show that *E. coli* TrmH does not require the interaction between D-loop and T-loop. This phenomenon is in line with the enzymatic property of *T. thermophilus* TrmH that *T. thermophilus* TrmH can methylate 5′-half fragment of *E. coli* tRNA^Met^_f_*(**62**)*. These results show that the basic tRNA recognition mechanism of *E. coli* TrmH is common with that of *T. thermophilus* TrmH *(**39**)*. In fact, the amino acid residues, which are important for tRNA recognition mechanism in *T. thermophilus* TrmH, are conserved in *E. coli* TrmH *(**46**)*.

### A single mutation (Glu107 to Gly) enabled crystallization of *E. coli* TrmH

To obtain structural information of *E. coli* TrmH, we attempted crystallization of the wild-type protein. However, this did not succeed because wild-type *E. coli* TrmH aggregated even in the presence of high concentrations of KCl. To overcome this problem, we compared the amino acid sequences of *E. coli* and *T. thermophilus* TrmH proteins *(**42**)* and identified several amino acid residues which are located on the surface of *T. thermophilus* TrmH and are not part of the SAM-binding site. Single mutations were introduced into *E. coli* TrmH and solubility of mutants checked. Fortunately, we found that one point mutant (Glu107 in *E. coli* TrmH was substituted by Gly; E107G) protein could be concentrated to more than 5 mg/ml in the presence of 200 mM KCl. Activity measurement of E107G mutant TrmH revealed that this mutant protein possesses comparable methyl-transfer activity to the wild-type enzyme (Supplementary Fig. S2A). Therefore, we used the E107G mutant TrmH for crystallization. This alteration enabled us to crystalize *E. coli* TrmH (Supplementary Fig. S2B).

### Overall structure of *E. coli* TrmH and differences to *T. thermophilus* TrmH

**Fig. 4:**
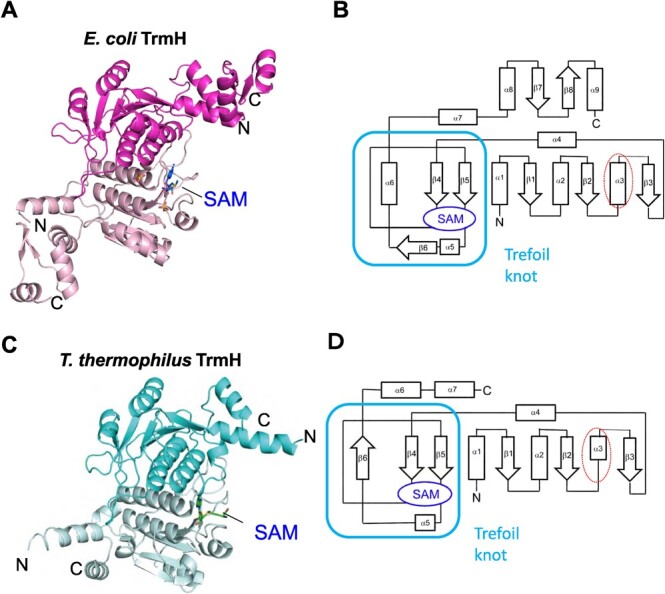
**Structure of *E. coli* TrmH.** (A) Dimer structure of *E. coli* TrmH is depicted as a cartoon model. The bound SAM in one subunit is highlighted as a stick model. N and C denote the N- and C-ends of the subunit, respectively. (B) Topology of *E. coli* TrmH is depicted. Arrows and boxes represent α-helices and β-strands, respectively. The long α3-helix is enclosed in a dotted circle. The location of SAM is shown by a circle and the trefoil knot region is enclosed. (C) Dimer structure of *T. thermophilus* TrmH is depicted in the same orientation as *E. coli* TrmH in panel A. The bound SAM in one subunit is highlighted as a stick model. N and C denote the N- and C-ends of the subunit, respectively. (D) Topology of *T. thermophilus* TrmH is depicted.

The structure of *E. coli* TrmH-SAM complex was solved at 1.95 Å resolution (Fig. 4A and Table 1, PDB code 7EDC). *E. coli* TrmH is a dimer and a trefoil knot structure is formed in each subunit (Fig. 4B). This is a typical feature of 2’-*O*-methyltransferases in the SPOUT superfamily *(**31**)*.

An obvious difference between *E. coli* (Fig. 4A and B) and *T. thermophilus* (Fig. 4C and D) TrmH proteins is the structure of C-terminal region. The C-terminal region of *E. coli* TrmH is longer than that of *T. thermophilus* and mainly forms α-helices as previously predicted by a bioinformatics study *(**45**)*, although one anti-parallel β-sheet (β7 and β8) is inserted (Fig. 4B). Our previous *in vitro* and *in vivo* study revealed that the C-terminal region is involved mainly in the initial binding of tRNA *(**48**)*. As shown in Fig. 2C, *E**. coli* TrmH bound not only to good substrate (tRNA^Ser^_CGA_) but also to poor substrate (tRNA^Phe^). Therefore, the difference in the C-terminal regions does not affect the selection of substrate tRNA directly.

**Fig. 5:**
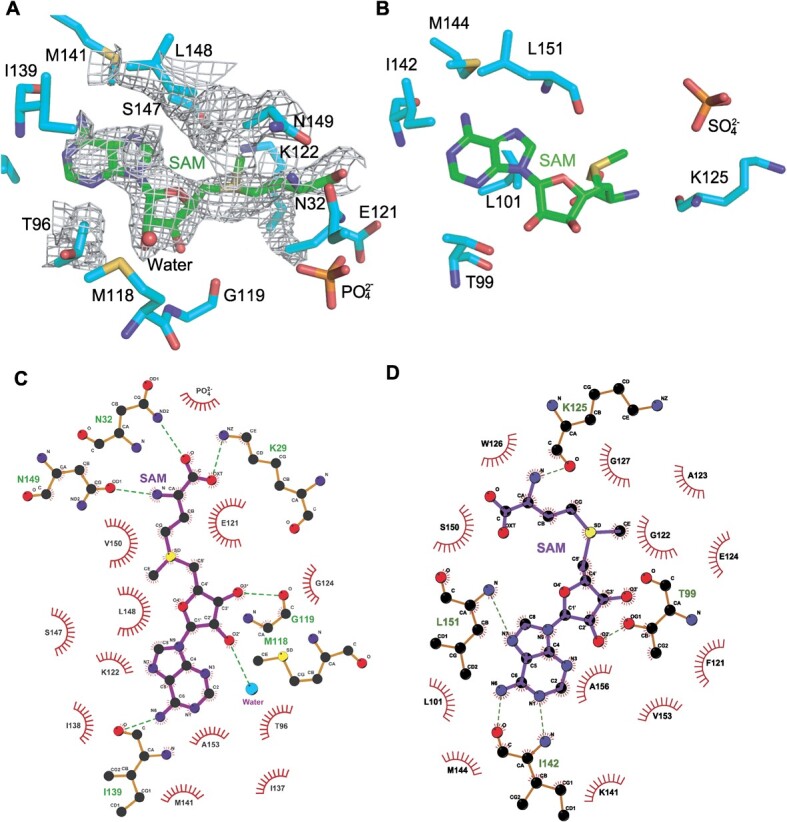
**SAM-binding pocket of *E. coli* and *T. thermophilus* TrmH proteins.** (A) Structure of SAM-binding pocket of *E. coli* TrmH is highlighted. Electron densities around SAM are hatched. C atoms in SAM and TrmH are indicated in green and cyan, respectively. O, N, S and P atoms are indicated in red, blue, yellow and orange, respectively. (B) Structure of SAM-binding pocket of *T. thermophilus* TrmH is highlighted. C atoms in SAM and TrmH are indicated in green and sky-blue, respectively. O, N, S and P atoms are indicated in red, blue, yellow and orange, respectively. (C) Interactions between amino acid residues in *E. coli* TrmH and bound SAM are shown. Dotted lines represent hydrogen bonds. (D) Interactions between amino acid residues in *T. thermophilus* TrmH and bound SAM are shown. Dotted lines represent hydrogen bonds.

A second difference is observed in the SAM-binding pocket (Fig. 5A). In comparison to the SAM-binding pocket of *T. thermophilus* TrmH (Fig. 5B) *(**35**)*, that of *E. coli* TrmH forms many hydrogen bonds with SAM. For example, the carboxyl group in SAM forms hydrogen bonds with Asn32 and Lys29 (Fig. 5C). These two residues are *E. coli* TrmH-specific and are not conserved in *T. thermophilus* TrmH (Fig. 5D) and other TrmH proteins. As a result, although the position of the methyl group in SAM is the same in *E. coli* and *T. thermophilus* TrmH proteins, the direction of the carboxyl group in SAM is different. *In vivo*, *T. thermophilus* TrmH acts at high temperatures (>50°C). Therefore, hydrophobic interactions may be more important than formation of hydrogen bonds for SAM-binding to *T. thermophilus* TrmH (Fig. 5B and5D).

Another difference in the catalytic domains of the two proteins can be observed. The polypeptide from Ser58 to Ser70 in *E. coli* TrmH forms a long α-helix (α3), which is shown enclosed in a dotted circle (Fig. 4B). In *T. thermophilus* TrmH, this region contains short α-helix and loop structure (Fig. 4D). This region is sandwiched between motifs 1 and 2 *(**30**,**46**)*, and there is no conserved amino acid residue among the TrmH family members. However, this long α-helix seems to bring the rigidity to the local structure of *E. coli* TrmH and may affect the methyl-transfer reaction. To investigate the role of the long α3-helix, we replaced this region of *E. coli* TrmH with that of *T. thermophilus* TrmH. However, this approach resulted in the mutant protein completely losing methyl-transfer activity (data not shown). In *E. coli* TrmH, loss of the long α3-helix may cause disruption of core structure of catalytic domain.

### The long variable region in good substrate tRNAs is required for efficient methylation by *E. coli* TrmH

**Fig. 6:**
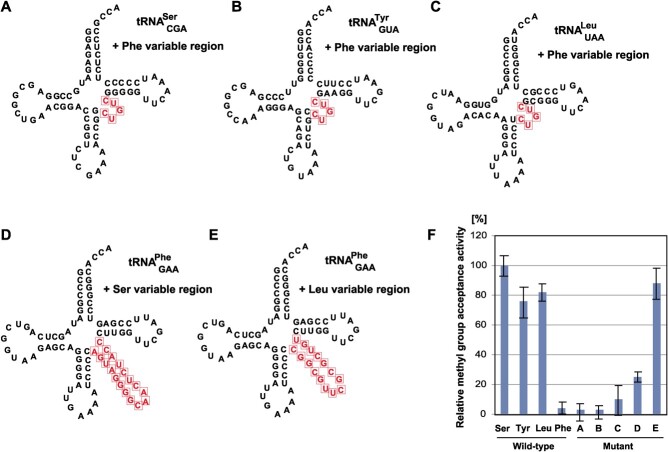
**The long variable region of good substrate (tRNA** ^**Ser**^_**CGA**_ **, tRNA** ^**Tyr**^_**GUA**_ **and tRNA** ^**Leu**^_**UAA**_ **) is required for the efficient methylation by *E. coli* TrmH.** The long variable region in tRNA^Ser^_CGA_ (A), tRNA^Tyr^_GUA_ (B) and tRNA^Leu^_UAA_ (C) were replaced with the regular size variable region of tRNA^Phe^. The regular size variable region in tRNA^Phe^ was replaced by the long variable region in tRNA^Ser^_CGA_ (D) and tRNA^Leu^_UAA_ (E). The mutation sites are enclosed by squares. (F) The methyl group acceptance activities of these mutant tRNA transcript (A-E) are compared to those of the wild-type tRNA transcripts. The methyl group acceptance activity of wild-type tRNA^Ser^_CGA_ is expressed as 100%.

As shown in Fig. 1A, tRNA^Ser^_CGA_, tRNA^Tyr^_GUA_ and tRNA^Leu^_CAG_ are relatively good substrates for *E. coli* TrmH. These tRNAs (so-called class II tRNAs) possess a long variable region (Fig. 6). In contrast, tRNA^Phe^ has a regular-size (5 nt) variable region and is a very poor substrate for *E. coli* TrmH. To clarify the role of long variable region, we prepared five mutant tRNA transcripts (Fig. 6A–E). When the variable region of tRNA^Ser^_CGA_ (Fig. 6A), tRNA^Tyr^_GUA_ (Fig. 6B) or tRNA^Leu^_UAA_ (Fig. 6C) was replaced with that of tRNA^Phe^, the methyl group acceptance activity dramatically decreased (Fig. 6F). In contrast, when the variable region of tRNA^Phe^ was replaced with that of tRNA^Ser^_CGA_ (Fig. 6D) or tRNA^Leu^_UAA_ (Fig. 6E), the methyl group acceptance activity clearly increased (Fig. 6F). Thus, these results show that the long variable region is important for efficient methylation by *E. coli* TrmH.

### The location of G18 in the D-loop is important for efficient methylation by *E. coli* TrmH

**Fig. 7:**
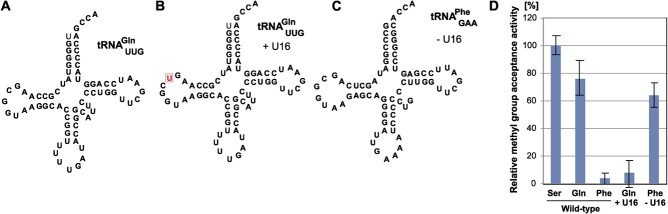
**The location of G18 in the D-loop was changed in good substrate (tRNA** ^**Gln**^ **) and very poor substrate (tRNA** ^**Phe**^ **).** (A) The structure of tRNA^Gln^ is depicted as a cloverleaf structure. (B) U16 was inserted into the D-loop of tRNA^Gln^ (good substrate). (C) U16 was deleted from the D-loop of tRNA^Phe^ (very poor substrate). (D) The methyl group acceptance activities of mutant tRNA transcript (B and C) are compared to those of the wild-type tRNA transcripts. The methyl group acceptance activity of wild-type tRNA^Ser^_CGA_ is expressed as 100%.

The long variable region is important for efficient methylation by *E. coli* TrmH. However, although tRNA^Gln^ has a regular size variable region (Fig. 7A), this tRNA possesses a Gm18 modification in living *E. coli* cells *(**7**)*. We noticed that the distance between G18 and A14 of tRNA^Gln^ is shorter than that in tRNA^Phe^ due to the deletion of one nucleotide (corresponding to pyrimidine 16 or 17). It should be mentioned that numbering of nucleotide positions in tRNA of this manuscript is according to Sprinzl *et al*. *(**63**)*. Therefore, in the case of tRNA^Gln^, one nucleotide is deleted between A14 and G18. Thus, this observation suggests that the location of G18 in the D-loop is important for efficient methylation by *E. coli* TrmH. To confirm this idea, we prepared two mutant tRNA transcripts (Fig. 7B and C) and their methyl group acceptance activities were tested (Fig. 7D). The wild-type tRNA^Gln^ transcript was methylated well by *E. coli* TrmH (Fig. 7D). However, the insertion of U16 resulted in a significant decrease of methyl group acceptance activity. Furthermore, the deletion of U16 in tRNA^Phe^ clearly increased methyl group acceptance activity. These results demonstrate that the location of G18 in the D-loop is important for efficient methylation by *E. coli* TrmH. Furthermore, because tRNA^Gln^ is methylated by *E. coli* TrmH, it is demonstrated that the long variable region itself is not essential: the long variable region may determine the location of G18 in the D-loop.

### Docking model of *E. coli* TrmH and tRNA

**Fig. 8:**
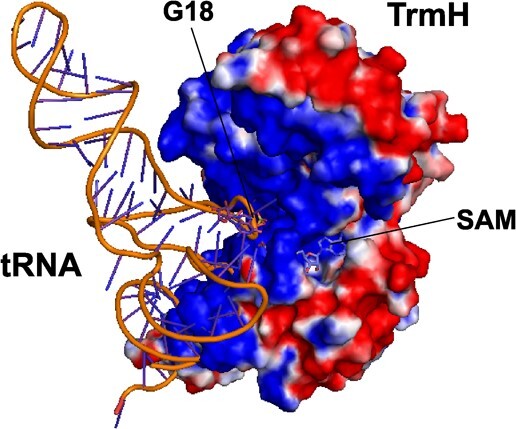
**Docking model of *E. coli* TrmH and tRNA complex.** Positive and negative charged surface areas of *E. coli* TrmH are colored in blue and red, respectively. The bound SAM is highlighted as a stick model. The structure of yeast tRNA^Phe^ is manually placed onto the surface of *E. coli* TrmH.

Finally, we constructed a docking model of *E. coli* TrmH and tRNA (Fig. 8). The positively charged surface area of *E. coli* TrmH is colored in blue. When the L-shaped tRNA was simply placed onto this area, crush of tRNA and TrmH structures occurred. Thus, to form the complex between tRNA and *E. coli* TrmH, structural changes of tRNA and protein are required. The location of G18 in the D-loop is very important for efficient methylation by *E. coli* TrmH. In the case of methylation of tRNA^Ser^, tRNA^Tyr^ and tRNA^Leu^, the D-loop structure produced by interaction with the long variable region is important. In the case of methylation of tRNA^Gln^, the short distance between G18 and A14 is important. These D-loop structures seem to be required for progress of the induced-fit process (the structural change process).

## Discussion

The *trmH* gene encoding tRNA (Gm18) methyltransferase in *E. coli* genome was initially predicted by a bioinformatics study in 1996 *(**18**)* and then confirmed by analysis of a gene disruption strain *(**19**)*. However, in more than two decades, the *trmH* gene product of *E. coli* has not been analyzed in terms of purified protein. To the authors’ knowledge, this is the first report of characterization of purified *E. coli* TrmH.

The enzymatic activity of *E. coli* TrmH is very unstable in the presence of 50 mM KCl (Supplementary Fig. S1). Therefore, at the start of this study, we searched a storage method for the protein. Fortunately, we found that a high concentration (400 mM) of KCl was effective and allowed storage of purified *E. coli* TrmH. The high concentrations of KCl may stabilize the dimer structure of *E. coli* TrmH, although we do not have the direct evidence. This feature may contribute to the regulation of enzymatic activity under the physiological conditions: *E. coli* TrmH rapidly loses the enzymatic activity after its expression in living cells. The second problem was that the activity of *E. coli* TrmH was very weak (Fig. 2). Our kinetic study has revealed that *E. coli* TrmH requires huge concentration of substrate tRNA for efficient methylation (Fig. 2A). Thus, under normal assay conditions, the activity of *E. coli* TrmH is barely detectable. This is consistent with the fact that *E. coli* TrmH activity was not detectable in cell extracts in an earlier study *(**64**)*. This low activity is not caused by the absence of other modifications of tRNA transcript. Indeed, the methylation speed of native tRNA^Ser^_CGA_ from the *ΔtrmH* strain is comparable to that of tRNA transcript (Fig. 1D). Thus, we have concluded that the low activity reflects the native characteristics of *E. coli* TrmH. In *E. coli*, D20a, ms^2^i^6^A37, m^5^U54 and Ψ55 in tRNA are a result of modifications by DusA *(**65**,**66**)*, MiaA *(**67**)* and MiaB *(**68**)*, TrmA *(**69**)* and TruB *(**70**)*, respectively. Although the presence of i^6^A37 modification by MiaA is essential for the 2’-*O*-methylation at position 34 by TrmL *(**44**,**71**)*, a member of SPOUT superfamily *(**72**)*, TrmH does not require this modification for the 2’-*O*-methylation of G18. It should be mentioned that the presence of modified nucleosides such as m^7^G46 (produced by TrmB) *(**73**,**74**)* in tRNA have effects on the methylation speed of *T. thermophilus* TrmH *(**12**)*. At high temperatures, modified nucleosides are required for the maintenance of L-shaped tRNA structure *(**75**)*. In contrast, Gm18 modification in *E. coli* tRNA is only required for survival in animal gut *(**14**,**15**)*. Therefore, *E. coli* TrmH seems to evolve to methylate a minimum subset of tRNAs to avoid consumption of excess amounts of SAM.

At 250 μM tRNA^Phe^ transcript, very slow methyl-transfer was observed. This concentration of single tRNA species is larger than that under physiological conditions in living *E. coli* cells. At high concentrations, tRNA^Phe^ that is not methylated under the physiological conditions is methylated. A similar phenomenon has been reported in *Saccharomyces cerevisiae (**76**)*. Trm10 is a member of SPOUT superfamily *(**77**)*, which produces the m^1^G9 modification in tRNA *(**78**)*. Overexpression of Trm10 in yeast yields m^1^G9 containing tRNA species that are ordinarily unmodified *in vivo (**76**)*. Thus, yeast Trm10 has a broader tRNA substrate specificity than is suggested by the observed pattern of modification in the yeast wild-type strain. Our previous *in vivo* experiment showed that overexpression of *E. coli* TrmH in *E. coli* cells increased the Gm content in tRNA fraction 1.7 fold *(**48**)*. Thus, this observation suggest that the Gm18 modification pattern in living cells is regulated by the balance between amounts of TrmH and substrate tRNA. In our previous study *(**40**)*, we classified TrmH enzymes simply based on their substrate tRNA specificities. As such, class I enzymes such as *T. thermophilus* TrmH methylate all tRNAs, whereas class II enzymes such as *E. coli* TrmH methylate a subset tRNAs. Our current study suggests that this classification might need to be reconsidered. *E. coli* TrmH has the potential to methylate tRNAs, which are not modified in physiological conditions.

The SAM-binding pocket (Fig. 5) of *E. coli* TrmH is slightly different from that of *T. thermophilus (**35**)* and *Aquifex aeolicus (**41**)* TrmH proteins. Notably, the carboxyl group in SAM forms hydrogen bonds with Asn32 and Lys29. Similar hydrogen bond formation is observed in the crystal structure of *E. coli* TrmJ and S-adenosyl-L-homocysteine *(**79**,**80**)*. TrmJ is a 2’-*O*-methyltransferase of the SPOUT superfamily and is responsible for the formation of Cm32 and Um32 in tRNA *(**79**,**80**)*. In the case of *E. coli* TrmJ, Arg23 forms a hydrogen bond with the carboxyl group of S-adenosyl-L-homocysteine: Arg23 is located between the N-terminal region and motif 1 and is not conserved in the SPOUT 2’-*O*-methyltransferases. Thus, the SAM-binding mode observed in the *E. coli* TrmH and SAM complex may be one variation of that seen in the SPOUT 2’-*O*-methyltransferases.

In living *E. coli* cells, only 14 tRNA species possess the Gm18 modification. For a long time, the question as to how *E. coli* TrmH selects specific tRNAs as substrates has remained unanswered. Our biochemical study has revealed that the D-loop structure by the interaction with long variable region in tRNA^Ser^_CGA_, tRNA^Tyr^_GUA_ and tRNA^Leu^_CAG_ is important for the efficient methylation by *E. coli* TrmH. Furthermore, in the case of tRNA^Gln^, the distance between G18 and A14 is important. These results demonstrate that the location of methylation site (2’-*O*-atom in G18) in the D-loop and deletion of a pyrimidine 17 is important for the efficient methylation by *E. coli* TrmH. To understand these issues structurally, structural studies of a complex of *E. coli* TrmH and tRNA is necessary.

### Accession number

The crystal structure factors and coordinates have been deposited in the Protein Data Bank (PDB code 7EDC).

## Supplementary Material

Web_Material_mvad076

## References

[1] Boccaletto, P., Machnicka, M.A., Purta, E., Piatkowski, P., Baginski, B., Wirecki, T.K., de Crécy-Lagard, V., Ross, R., Limbach, P.A., Kotter, A., Helm, M., and Bujnicki, J.M. (2018) MODOMICS: a database of RNA modification pathways. 2017 update. Nucleic Acids Res. 46, D303–D307 29106616 10.1093/nar/gkx1030 PMC5753262

[2] Lorenz, C., Lünse, C.E., and Mörl, M. (2017) tRNA modifications: impact on structure and thermal adaptation. Biomol. Ther. 7, 35 10.3390/biom7020035 PMC5485724 28375166

[3] Väre, V.Y., Eruysal, E.R., Narendran, A., Sarachan, K.L., and Agris, P.F. (2017) Chemical and conformational diversity of modified nucleosides affects tRNA structure and function. Biomol. Ther. 7, 29 10.3390/biom7010029 PMC5372741 28300792

[4] Hori, H. (2014) Methylated nucleosides in tRNA and tRNA methyltransferases. Front. Genet. 5, 144 24904644 10.3389/fgene.2014.00144 PMC4033218

[5] Kawai, G., Yamamoto, Y., Kamimura, T., Masegi, T., Sekine, M., Hata, T., Iimori, T., Watanabe, T., Miyazawa, T., and Yokoyama, S. (1992) Conformational rigidity of specific pyrimidine residues in tRNA arises from posttranscriptional modifications that enhance steric interaction between the base and the 2′-hydroxyl group. Biochemistry 31, 1040–1046 1310418 10.1021/bi00119a012

[6] Kumagai, I., Watanabe, K., and Oshima, T. (1982) A thermostable tRNA (guanosine-2′)-methyltransferase from *Thermus thermophilus* HB27 and the effect of ribose methylation on the conformational stability of tRNA. J. Biol. Chem. 257, 7388–7395 7085632

[7] Jühling, F., Mörl, M., Hartmann, R.K., Sprinzl, M., Stadler, P.F., and Pütz, J. (2009) tRNAdb 2009: compilation of tRNA sequences and tRNA genes. Nucleic Acids Res. 37, D159–D162 18957446 10.1093/nar/gkn772 PMC2686557

[8] Robertus, J.D., Ladner, J.E., Finch, J.T., Rhodes, D., Brown, R.S., Clark, B.F., and Klug, A. (1974) Structure of yeast phenylalanine tRNA at 3 A resolution. Nature 250, 546–551 4602655 10.1038/250546a0

[9] Kim, S.H., Sussman, J.L., Suddath, F.L., Quigley, G.J., McPherson, A., Wang, A.H., Seeman, N.C., and Rich, A. (1974) The general structure of transfer RNA molecules. Proc. Natl. Acad. Sci. U. S. A. 71, 4970–4974 4612535 10.1073/pnas.71.12.4970 PMC434021

[10] Motorin, Y. and Helm, M. (2010) tRNA stabilization by modified nucleotides. Biochemistry 49, 4934–4944 20459084 10.1021/bi100408z

[11] Roovers, M., Droogmans, L., and Grosjean, H. (2021) Post-transcriptional modifications of conserved nucleotides in the T-loop of tRNA: a tale of functional convergent evolution. Genes 12, 140 33499018 10.3390/genes12020140 PMC7912444

[12] Tomikawa, C., Yokogawa, T., Kanai, T., and Hori, H. (2010) *N*^7^-methylguanine at position 46 (m^7^G46) in tRNA from *Thermus thermophilus* is required for cell viability through a tRNA modification network. Nucleic Acids Res. 38, 942–957 19934251 10.1093/nar/gkp1059 PMC2817472

[13] Ishida, K., Kunibayashi, T., Tomikawa, C., Ochi, A., Kanai, T., Hirata, A., Iwashita, C., and Hori, H. (2011) Pseudouridine at position 55 in tRNA controls the contents of other modified nucleotides for low-temperature adaptation in the extreme-thermophilic eubacterium *Thermus thermophilus*. Nucleic Acids Res. 39, 2304–2318 21097467 10.1093/nar/gkq1180 PMC3064792

[14] Takuma, H., Ushio, N., Minoji, M., Kazayama, A., Shigi, N., Hirata, A., Tomikawa, C., Ochi, A., and Hori, H. (2015) Substrate tRNA recognition mechanism of eubacterial tRNA (m^1^A58) methyltransferase (TrmI). J. Biol. Chem. 290, 5912–5925 25593312 10.1074/jbc.M114.606038 PMC4342497

[15] Yamagami, R., Tomikawa, C., Shigi, N., Kazayama, A., Asai, S., Takuma, H., Hirata, A., Fourmy, D., Asahara, H., Watanabe, K., Yoshizawa, S., and Hori, H. (2016) Folate-/FAD-dependent tRNA methyltransferase from *Thermus thermophilus* regulates other modifications in tRNA at low temperatures. Genes Cells 21, 740–754 27238446 10.1111/gtc.12376

[16] Hori, H. (2019) Regulatory factors for tRNA modifications in extreme-thermophilic bacterium *Thermus thermophilus*. Front. Genet. 10, 204 30906314 10.3389/fgene.2019.00204 PMC6418473

[17] Gehrig, S., Eberle, M.-E., Botschen, F., Rimbach, K., Eberle, F., Eigenbrod, T., Kaiser, S., Holmes, W.M., Erdmann, V.A., Sprinzl, M., Bec, G., Keith, G., Dalpke, A.H., and Helm, M. (2012) Identification of modifications in microbial, native tRNA that suppress immunostimulatory activity. J. Exp. Med. 209, 225–233 22312113 10.1084/jem.20111044 PMC3280868

[18] Jöckel, S., Nees, G., Sommer, R., Zhao, Y., Cherkasov, D., Hori, H., Ehm, G., Schnare, M., Nain, M., Kaufmann, A., and Bauer, S.T. (2012) The 2′-O-methylation status of a single guanosine controls transfer RNA–mediated toll-like receptor 7 activation or inhibition. J. Exp. Med. 209, 235–241 22312111 10.1084/jem.20111075 PMC3280869

[19] Freund, I., Buhl, D.K., Boutin, S., Kotter, A., Pichot, F., Marchand, V., Vierbuchen, T., Heine, H., Motorin, Y., Helm, M., Dalpke, A.H., and Eigenbrod, T. (2019) 2'-*O*-methylation within prokaryotic and eukaryotic tRNA inhibits innate immune activation by endosomal toll-like receptors but does not affect recognition of whole organisms. RNA 25, 869–880 31019095 10.1261/rna.070243.118 PMC6573781

[20] Galvanin, A., Vogt, L.M., Grober, A., Freund, I., Ayadi, L., Bourguignon-Igel, V., Bessler, L., Jacob, D., Eigenbrod, T., Marchand, V., Dalpke, A., Helm, M., and Motorin, Y. (2020) Bacterial tRNA 2'-*O*-methylation is dynamically regulated under stress conditions and modulates innate immune response. Nucleic Acids Res. 48, 12833–12844 33275131 10.1093/nar/gkaa1123 PMC7736821

[21] Gustafsson, C., Reid, R., Greene, P.J., and Santi, D.V. (1996) Identification of new RNA modifying enzymes by iterative genome search using known modifying enzymes as probes. Nucleic Acids Res. 24, 3756–3762 8871555 10.1093/nar/24.19.3756 PMC146159

[22] Persson, B.C., Jäger, G., and Gustafsson, C. (1997) The spoU gene of *Escherichia coli*, the fourth gene of the spot operon, is essential for tRNA (Gm18) 2’-*O*-methyltransferase activity. Nucleic Acid Res. 25, 4093–4097 9321663 10.1093/nar/25.20.4093 PMC146995

[23] Hori, H., Suzuki, T., Sugawara, K., Inoue, Y., Shibata, T., Kuramitsu, S., Yokoyama, S., Oshima, T., and Watanabe, K. (2002) Identification and characterization of tRNA (Gm18) methyltransferase from *Thermus thermophilus* HB8: domain structure and conserved amino acid sequence motifs. Genes Cells 7, 259–272 11918670 10.1046/j.1365-2443.2002.00520.x

[24] Cavaillé, J., Chetouani, F., and Bachellerie, J.-P. (1999) The yeast *Saccharomyces cerevisiae* YDL112w ORF encodes the putative 2’-O-ribose methyltransferase catalyzing the formation of Gm18 in tRNAs. RNA 5, 66–81 9917067 10.1017/s1355838299981475 PMC1369740

[25] Wu, F., Garcia, J., Sigman, D., and Gaynor, R. (1991) Tat regulates binding of the human immunodeficiency virus trans-activating region RNA loop-binding protein TRP-185. Genes Dev. 5, 2128–2140 1936997 10.1101/gad.5.11.2128

[26] Kiss-László, Z., Henry, Y., Bachellerie, J.P., Caizergues-Ferrer, M., and Kiss, T. (1996) Site-specific ribose methylation of preribosomal RNA: a novel function for small nucleolar RNAs. Cell 85, 1077–1088 8674114 10.1016/s0092-8674(00)81308-2

[27] Omer, A.D., Zago, M., Chang, A., and Dennis, P.P. (2006) Probing the structure and function of an archaeal C/D-box methylation guide sRNA. RNA 12, 1708–1720 16861619 10.1261/rna.31506 PMC1557695

[28] Wolff, P., Villette, C., Zumsteg, J., Heintz, D., Antoine, L., Chane-Woon-Ming, B., Droogmans, L., Grosjean, H., and Westhof, E. (2020) Comparative patterns of modified nucleotides in individual tRNA species from a mesophilic and two thermophilic archaea. RNA 26, 1957–1975 32994183 10.1261/rna.077537.120 PMC7668247

[29] Schubert, H.G., Blumenthal, R.M., and Cheng, X. (2003) Many paths to methyltransfer: a chronicle of convergence. Trends Biochem. Sci. 28, 329–335 12826405 10.1016/S0968-0004(03)00090-2 PMC2758044

[30] Anantharaman, V., Koonin, E.V., and Aravind, L. (2002) SPOUT: a class of methyltransferases that includes *spoU* and *trmD* RNA methylase superfamilies, and novel superfamilies of predicted prokaryotic RNA methylases. J. Mol. Microbiol. Biotechnol. 4, 71–75 11763972

[31] Hori, H. (2017) Transfer RNA methyltransferases with a SpoU-TrmD (SPOUT) fold and their modified nucleosides in tRNA. Biomol. Ther. 7, 23

[32] Krishnamohan, A. and Jackman, J.E. (2019) A family divided: distinct structural and mechanistic features of the SpoU-TrmD (SPOUT) methyltransferase superfamily. Biochemistry 58, 336–345 30457841 10.1021/acs.biochem.8b01047 PMC6541868

[33] Byström, A.S. and Björk, G.R. (1982) Chromosomal location and cloning of the gene (trmD) responsible for the synthesis of tRNA (m^1^G) methyltransferase in *Escherichia coli* K-12. Mol. Gen. Genet. 188, 440–446 6298573 10.1007/BF00330046

[34] Hou, Y.M., Matsubara, R., Takase, R., Masuda, I., and Sulkowska, J.I. (2017) TrmD: a methyl transferase for tRNA methylation with m^1^G37. Enzymes 41, 89–115 28601227 10.1016/bs.enz.2017.03.003 PMC6054489

[35] Nureki, O., Watanabe, K., Fukai, S., Ishii, R., Endo, Y., Hori, H., and Yokoyama, S. (2004) Deep knot structure for construction of active site and cofactor binding site of tRNA modification enzyme. Structure 12, 593–602 15062082 10.1016/j.str.2004.03.003

[36] Ahn, H.J., Kim, H.W., Yoon, H.J., Lee, B., Suh, S.S., and Yang, J.K. (2003) Crystal structure of tRNA (m^1^G37) methyltransferase: insights into tRNA recognition. EMBO J. 22, 2593–2603 12773376 10.1093/emboj/cdg269 PMC156765

[37] Elkins, P.A., Watts, J.M., Zalacain, M., van Thiel, A., Vitazka, P.R., Redlak, M., Andraos-Selim, C., Rastinejad, F., and Holmes, W.M. (2003) Insights into catalysis by a knotted TrmD tRNA methyltransferase. J. Mol. Biol. 333, 931–949 14583191 10.1016/j.jmb.2003.09.011

[38] Liu, J., Wang, W., Shin, D.H., Yokota, H., Kim, R., and Kim, S.-H. (2003) Crystal structure of tRNA (m^1^G37) methyltransferase from *Aquifex aeolicus* at 2.6 a resolution: a novel methyltransferase fold. Proteins 53, 326–328 14517984 10.1002/prot.10479

[39] Hori, H., Yamazaki, N., Matsumoto, T., Watanabe, Y., Ueda, T., Nishikawa, K., Kumagai, I., and Watanabe, K. (1998) Substrate recognition of tRNA (Guanosine-2′-)-methyltransferase from *Thermus thermophilus* HB27. J. Biol. Chem. 273, 25721–25727 9748240 10.1074/jbc.273.40.25721

[40] Hori, H., Kubota, S., Watanabe, K., Kim, J.M., Ogasawara, T., Sawasaki, T., and Endo, Y. (2003) *Aquifex aeolicus* tRNA (Gm18) methyltransferase has unique substrate specificity. J. Biol. Chem. 278, 25081–25090 12704200 10.1074/jbc.M212577200

[41] Pleshe, E., Truesdell, J., and Batey, R.T. (2005) Structure of a class II TrmH tRNA-modifying enzyme from *Aquifex aeolicus*. Acta Crystallogr. Sect. F Struct. Biol. Cryst. Commun. 61, 722–728 10.1107/S1744309105022980 PMC1952360 16511140

[42] Watanabe, K., Nureki, O., Fuakai, S., Ishii, R., Okamoto, H., Yokoyama, S., Endo, Y., and Hori, H. (2005) Roles of conserved amino acid sequence motifs in the SpoU (TrmH) RNA methyltransferase family. J. Biol. Chem. 280, 10368–10377 15637073 10.1074/jbc.M411209200

[43] Kuratani, M., Bessho, Y., Nishimoto, M., Grosjean, H., and Yokoyama, S. (2008) Crystal structure and mutational study of a unique SpoU family archaeal methylase that forms 2’-*O*-methylcytidine at position 56 of tRNA. J. Mol. Biol. 375, 1064–1075 18068186 10.1016/j.jmb.2007.11.023

[44] Liu, R.J., Zhou, M., Fang, Z.P., Wang, M., Zhou, X.L., and Wang, E.D. (2013) The tRNA recognition mechanism of the minimalist SPOUT methyltransferase, TrmL. Nucleic Acids Res. 41, 7828–7842 23804755 10.1093/nar/gkt568 PMC3763551

[45] Tkaczuk, K.L., Dunin-Horkawicz, S., Purta, E., and Bujnicki, J.M. (2007) Structural and evolutionary bioinformatics of the SPOUT superfamily of methyltransferases. BMC Bioinformatics 8, 73 17338813 10.1186/1471-2105-8-73 PMC1829167

[46] Watanabe, K., Nureki, O., Fukai, S., Endo, Y., and Hori, H. (2006) Functional categorization of the conserved basic amino acid residues in TrmH (tRNA(Gm18)methyltransferase) enzymes. J. Biol. Chem. 281, 34630–34639 16963456 10.1074/jbc.M606141200

[47] Ochi, A., Makabe, K., Kuwajima, K., and Hori, H. (2010) Flexible recognition of the tRNA G18 methylation target site by TrmH methyltransferase through first binding and induced fit processes. J. Biol. Chem. 285, 9018–9029 20053984 10.1074/jbc.M109.065698 PMC2838323

[48] Ochi, A., Makabe, K., Yamagami, R., Hirata, A., Sakaguchi, R., Hou, Y.M., Watanabe, K., Nureki, O., Kuwajima, K., and Hori, H. (2013) The catalytic domain of topological knot tRNA methyltransferase (TrmH) discriminates between substrate tRNA and nonsubstrate tRNA *via* an induced-fit process. J. Biol. Chem. 288, 25562–25574 23867454 10.1074/jbc.M113.485128 PMC3757217

[49] Hori, H. (2010) Synthesis of a hetero subunit RNA modification enzyme by the wheat germ cell-free translation system. Methods Mol. Biol. 607, 173–185 20204857 10.1007/978-1-60327-331-2_15

[50] Baba, T., Ara, T., Hasegawa, M., Takai, Y., Okumura, Y., Baba, M., Datsenko, K.A., Tomita, M., Wanner, B.L., and Mori, H. (2006) Construction of Escherichia coli K-12 in-frame, single-gene knockout mutants: the Keio collection. Nol. Syst. Biol. 2, 0008 10.1038/msb4100050 PMC1681482 16738554

[51] Chomezynski, P. and Sacchi, N. (1987) Single-step method of RNA isolation by acid guanidinium thiocyanate-phenol-chloroform extraction. Anal. Biochem. 162, 156–159 2440339 10.1006/abio.1987.9999

[52] Yokogawa, T., Kitamura, Y., Nakamura, D., Ohno, S., and Nishikawa, K. (2010) Optimization of the hybridization-based method for purification of thermostable tRNAs in the presence of tetraalkylammonium salts. Nucleic Acids Res. 38, e89 20040572 10.1093/nar/gkp1182 PMC2847242

[53] Kazayama, A., Yamagami, R., Yokogawa, T., and Hori, H. (2015) Improved solid-phase DNA probe method for tRNA purification: large scale preparation, and alteration of DNA fixation. J. Biochem. 157, 411–418 25572528 10.1093/jb/mvu089

[54] Laskowski, R., MacArthur, M., Moss, D., and Thoronton, J. (1992) PROCHECK: a program to check the stereochemical quality ofprotein structures. J. Appl. Crystallogr. 26, 283–291

[55] Otwinowski, Z. and Minor, W. (1997) Processing of X-ray diffraction data collected in oscillation mode. Methods Enzymol. 276, 307–326 27754618 10.1016/S0076-6879(97)76066-X

[56] Adams, P., Grosse-Kunstleve, R., Hung, L., Ioerger, T., McCoy, A., Moriarty, N., Read, R., Sacchettini, J., Sauter, N., and Terwilliger, T. (2002) PHENIX: building new software for automated crystallographic structure determination. Acta Crystallogr D Biol. Crystallogr. 58, 1948–1954 12393927 10.1107/s0907444902016657

[57] Terwilliger, T.C. (2003) Automated side-chain model building and sequence assignment by template matching. Acta Crystallogr D Biol. Crystallogr. 59, 45–49 12499538 10.1107/S0907444902018048 PMC2745879

[58] Emsley, P. and Cowtan, K. (2004) Coot: model-building tools for molecular graphics. Acta Crystallogr D Biol. Crystallogr. 60, 2126–2132 15572765 10.1107/S0907444904019158

[59] McCoy, A., Grosse-Kunstleve, R., Adams, P., Winn, M., Storoni, L., and Read, R. (2007) Phaser crystallographic software. J. Appl. Crystallogr. 40, 658–674 19461840 10.1107/S0021889807021206 PMC2483472

[60] Murashudov, N.G., Skubák, P., Lebedev, A., Pannu, S.N., Steiner, R.A., Nicholls, R.A., Winn, D.M., Long, F., and Vagin, A.A. (2011) REFMAC5 for the refinement of macromolecular crystal structures. Acta Crystallogr D Biol. Crystallogr. 67, 355–367 21460454 10.1107/S0907444911001314 PMC3069751

[61] Hori, H., Terui, Y., Nakamoto, C., Iwashita, C., Ochi, A., Watanabe, K., and Oshima, T. (2016) Effects of polyamines from *Thermus thermophilus*, an extreme-thermophilic eubacterium, on tRNA methylation by tRNA (Gm18) methyltransferase (TrmH). J. Biochem. 159, 509–517 26721905 10.1093/jb/mvv130

[62] Matsumoto, T., Nishikawa, K., Hori, H., Ohta, T., Miura, K., and Watanabe, K. (1990) Recognition sites of tRNA by a thermostable tRNA(guanosine-2′-)-methyltransferase from *Thermus thermophilus* HB27. J. Biochem. 107, 331–338 2187856 10.1093/oxfordjournals.jbchem.a123047

[63] Sprinzl, M., Horn, C., Brown, M., Ioudovitch, A., and Steinberg, S. (1998) Compilation of tRNA sequences and sequences of tRNA genes. Nucleic Acids Res. 26, 148–153 9399820 10.1093/nar/26.1.148 PMC147216

[64] Hurwitz, J., Gold, M., and Anders, M. (1964) The enzymatic methylation of ribonucleic acid and deoxyribonucleic acid III. Purification of soluble ribonucleic acid methylating enzymes. J. Biol. Chem. 239, 3462–3473 14245404

[65] Bishop, A.C., Xu, J., Johnson, R.C., Schimmel, P., and de Crécy-Lagard, V. (2002) Identification of the tRNA-dihydrouridine synthase family. J. Biol. Chem. 277, 25090–25095 11983710 10.1074/jbc.M203208200

[66] Bou-Nader, C., Montémont, H., Guérineau, V., Jean-Jean, O., Brégeon, D., and Hamdane, D. (2018) Unveiling structural and functional divergences of bacterial tRNA dihydrouridine synthases: perspectives on the evolution scenario. Nucleic Acids Res. 46, 1386–1394 29294097 10.1093/nar/gkx1294 PMC5814906

[67] Caillet, J. and Droogmans, L. (1988) Molecular cloning of the *Escherichia coli miaA* gene involved in the formation of delta 2-isopentenyl adenosine in tRNA. J. Bacteriol. 170, 4147–4152 3045085 10.1128/jb.170.9.4147-4152.1988 PMC211421

[68] Arragain, S., Handelman, S.K., Forouhar, F., Wei, F.Y., Tomizawa, K., Hunt, J.F., Douki, T., Fontecave, M., Mulliez, E., and Atta, M. (2010) Identification of eukaryotic and prokaryotic methylthiotransferase for biosynthesis of 2-methylthio-N6-threonylcarbamoyladenosine in tRNA. J. Biol. Chem. 285, 28425–28433 20584901 10.1074/jbc.M110.106831 PMC2937867

[69] Ny, T. and Björk, G.R. (1980) Cloning and restriction mapping of the *trmA* gene coding for transfer ribonucleic acid (5-methyluridine)-methyltransferase in *Escherichia coli* K-12. J. Bacteriol. 142, 371–379 6247318 10.1128/jb.142.2.371-379.1980 PMC293980

[70] Nurse, K., Wrzesinski, J., Bakin, A., Lane, B.G., and Ofengand, J. (1995) Purification, cloning, and properties of the tRNA psi 55 synthase from *Escherichia coli*. RNA 1, 102–112 7489483 PMC1369054

[71] Benítez-Páez, A., Villarroya, M., Douthwaite, S., Gabaldón, T., and Armengod, M.E. (2010) YibK is the 2'-*O*-methyltransferase TrmL that modifies the wobble nucleotide in *Escherichia coli* tRNA(Leu) isoacceptors. RNA 16, 2131–2143 20855540 10.1261/rna.2245910 PMC2957053

[72] Lim, K., Zhang, H., Tempczyk, A., Krajewski, W., Bonander, N., Toedt, J., Howard, A., Eisenstein, E., and Herzberg, O. (2003) Structure of the YibK methyltransferase from *Haemophilus influenzae* (HI0766): a cofactor bound at a site formed by a knot. Proteins 51, 56–67 12596263 10.1002/prot.10323

[73] De Bie, L.G., Roovers, M., Oudjama, Y., Wattiez, R., Tricot, C., Stalon, V., Droogmans, L., and Bujnicki, J.M. (2003) The *yggH* gene of *Escherichia coli* encodes a tRNA (m^7^G46) methyltransferase. J. Bacteriol. 185, 3238–3243 12730187 10.1128/JB.185.10.3238-3243.2003 PMC154064

[74] Okamoto, H., Watanabe, K., Ikeuchi, Y., Suzuki, T., Endo, Y., and Hori, H. (2004) Substrate tRNA recognition mechanism of tRNA (m^7^G46) methyltransferase from *Aquifex aeolicus*. J. Biol. Chem. 279, 49151–49159 15358762 10.1074/jbc.M408209200

[75] Hori, H., Kawamura, T., Awai, T., Ochi, A., Yamagami, R., Tomikawa, C., and Hirata, A. (2018) Transfer RNA modification enzymes from thermophiles and their modified nucleosides in tRNA. Microorganisms 6, 110 30347855 10.3390/microorganisms6040110 PMC6313347

[76] Swinehart, W.E., Henderson, J.C., and Jackman, J.E. (2013) Unexpected expansion of tRNA substrate recognition by the yeast m^**1**^G9 methyltransferase Trm10. RNA 19, 1137–1146 23793893 10.1261/rna.039651.113 PMC3708533

[77] Shao, Z., Yan, W., Peng, J., Zuo, X., Zou, Y., Li, F., Gong, D., Ma, R., Wu, J., Shi, Y., Zhang, Z., Teng, M., Li, X., and Gong, Q. (2014) Crystal structure of tRNA m^**1**^G9 methyltransferase Trm10: insight into the catalytic mechanism and recognition of tRNA substrate. Nucleic Acids Res. 42, 509–525 24081582 10.1093/nar/gkt869 PMC3874184

[78] Jackman, J.E., Montange, R.K., Malik, H.S., and Phizicky, E.M. (2003) Identification of the yeast gene encoding the tRNA m^1^G methyltransferase responsible for modification at position 9. RNA 9, 574–585 12702816 10.1261/rna.5070303 PMC1370423

[79] Purta, E., van Vliet, F., Tkaczuk, K.L., Dunin-Horkawicz, S., Mori, H., Droogmans, L., and Bujnicki, J.M. (2006) The *yfhQ* gene of *Escherichia coli* encodes a tRNA:Cm32/Um32 methyltransferase. BMC Mol. Biol. 7, 23 16848900 10.1186/1471-2199-7-23 PMC1569432

[80] Somme, J., Van Laer, B., Roovers, M., Steyaert, J., Versées, W., and Droogmans, L. (2014) Characterization of two homologous 2'-*O*-methyltransferases showing different specificities for their tRNA substrates. RNA 20, 1257–1271 24951554 10.1261/rna.044503.114 PMC4105751

