# Supplementary material for: Escherichia coli tRNA (Gm18) methyltransferase (TrmH) requires the correct localization of its methylation site (G18) in the D-loop for efficient methylation
Source: J Biochem. 2023 Oct 16;175(1):43–56. doi: 10.1093/jb/mvad076 (PMC11640301; doi:10.1093/jb/mvad076)
Supplement: Web_Material_mvad076 [file web_material_mvad076.pdf]

**Supporting information**

# *Escherichia coli* tRNA (Gm18) methyltransferase, TrmH requires the correct localization of its methylation site (G18) in the D-loop for efficient methylation.

**Yoh Kohno[1*], Asako Ito[1*], Aya Okamoto[1], Ryota Yamagami[1], Akira Hirata[2, **], and Hiroyuki Hori[1, **]**

[1]Department of Materials Science and Biotechnology, Graduate school of Science and Engineering, Ehime University, 3 Bunkyo-cho, Matsuyama, Ehime 790-8577, Japan

[2]Department of Natural Science, Graduate School of Technology, Industrial and Social Science, Tokushima University, 2-1 Minamijosanjimacho, Tokushima, Tokushima 770-8506, Japan

*These authors equally contributed to this work.

**To whom correspondence should be addressed:

Hiroyuki Hori

Department of Materials Science and Biotechnology, Graduate School of Science and Engineering, Ehime University, 3 Bunkyo-cho, Matsuyama, Ehime 790-8577, Japan.

Phone: 81-89-927-8548

E-mail: hori.hiroyuki.my@ehime-u.ac.jp

Akira Hirata

Department of Natural Science, Graduate School of Technology,

Industrial and Social Science, Tokushima University, 2-1 Minamijosanjima-cho, Tokushima, Tokushima, 770-8506, Japan.

Phone: 81-88-656-7261

E-mail: ahirata@tokushima-u.ac.jp

Running title: tRNA recognition mechanism of *E. coli* TrmH

Abbreviations: Gm, 2'-*O*-methylguanosine; Ψ, pseudouridine

**Legends to Supporting Figures**

**SFig. 1. A high concentration of KCl is required for the storage of active purified TrmH.** (A) Purified *E. coli* TrmH (4 μg) was analyzed by 15% SDS-PAGE. The gel was stained with Coomassie Brilliant Blue. (B) Purified *E. coli* TrmH (500 μg/ml) was stored in buffer [50 mM Tris-HCl (pH 7.6), 5 mM $MgCl_2$, 6 mM 2-mercaptoethanol,50 mM KCl and 5% glycerol] at 4°C. The filled circles show the methylation activity of purified TrmH before storage. After 14 days, the methylation activity was decreased (open circles). (C) When the *E. coli* TrmH (500 μg/ml) was stored in buffer [50 mM Tris-HCl (pH 7.6), 5 mM $MgCl_2$, 6 mM 2-mercaptoethanol,400 mM KCl and 50% glycerol] for 14 days, the methylation activity (open circles) did not change significantly compared to the activity before storage (filled circles).

**SFig. 2.** (A) The methyl-transfer activities of wild-type and E107G mutant TrmH were measured. 100 μM $tRNA^{Ser}_{CGA}$ transcript was used as the substrate. (B) Crystals of *E. coli* E107G mutant TrmH. In the first screening, these crystals were obtained. The bar represents 0.1 mm length. Based on this pilot experiment, conditions for crystallization were analyzed.

SFig. 1

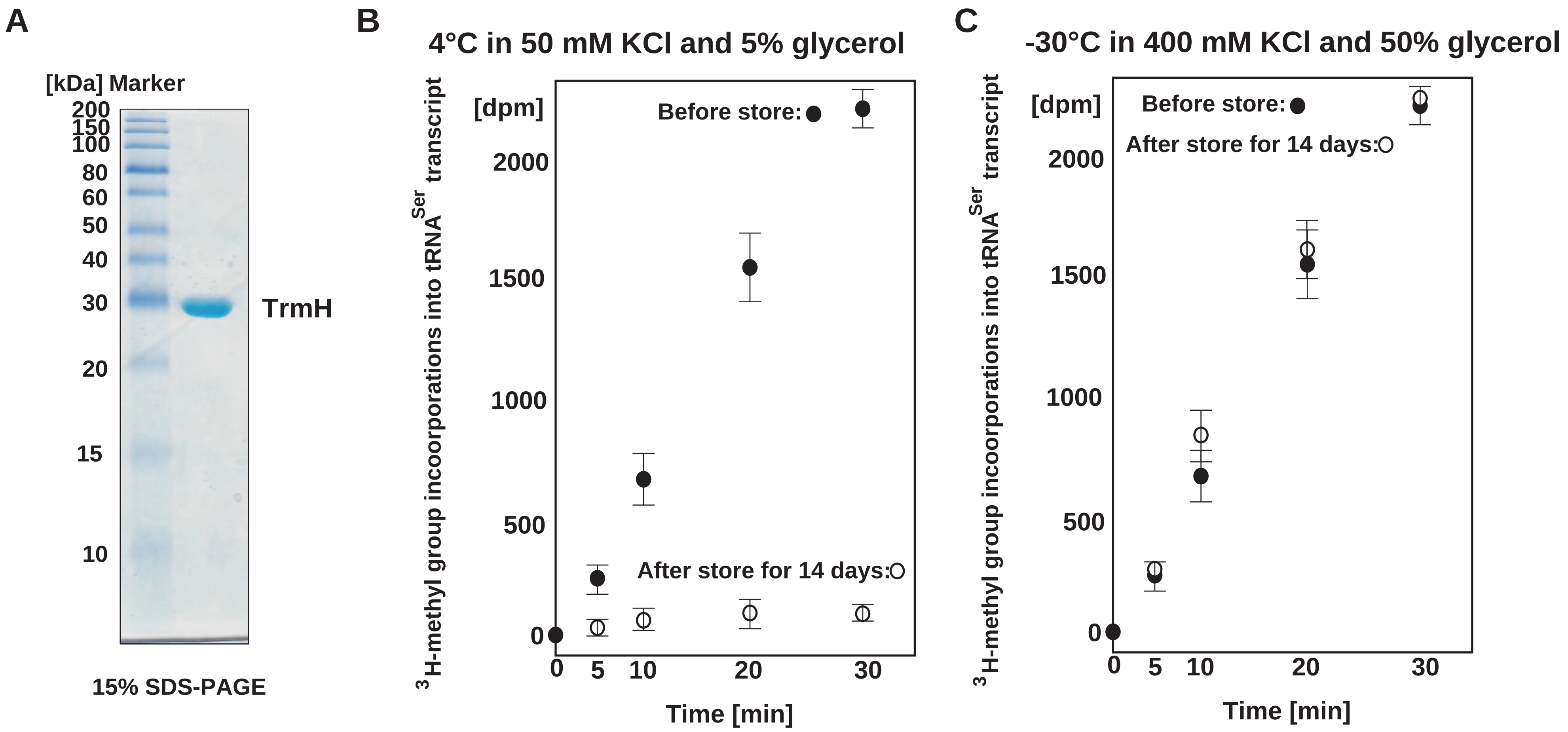
A
[kDa] Marker
200
150
100
80
60
50
40
30
20
15
10
TrmH
15% SDS-PAGE
B
4°C in 50 mM KCl and 5% glycerol
[dpm]
Before store:
After store for 14 days:
2000
1500
1000
500
0
0
5
10
20
30
Time [min]
3H-methyl group incoorporations into tRNASer transcript
C
-30°C in 400 mM KCl and 50% glycerol
[dpm]
Before store:
After store for 14 days:
2000
1500
1000
500
0
0
5
10
20
30
Time [min]
3H-methyl group incoorporations into tRNASer transcript

SFig. 2

A

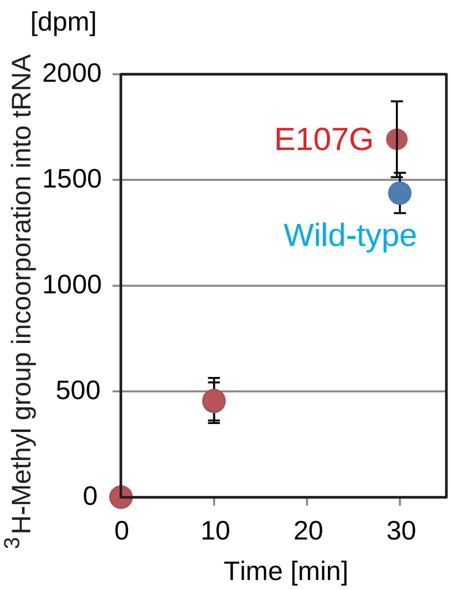

[dpm]
$^{3}$H-Methyl group incoorporation into tRNA
2000
1500
1000
500
0
0
10
20
30
Time [min]
E107G
Wild-type


B

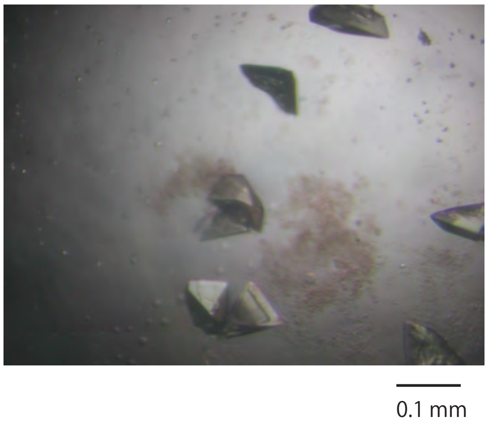

0.1 mm
